# It Takes Two: Dimerization Is Essential for the Broad-Spectrum Predatory and Defensive Activities of the Venom Peptide Mp1a from the Jack Jumper Ant *Myrmecia pilosula*

**DOI:** 10.3390/biomedicines8070185

**Published:** 2020-06-30

**Authors:** Samantha A. Nixon, Zoltan Dekan, Samuel D. Robinson, Shaodong Guo, Irina Vetter, Andrew C. Kotze, Paul F. Alewood, Glenn F. King, Volker Herzig

**Affiliations:** 1 Institute for Molecular Bioscience, The University of Queensland, St Lucia, QLD 4072, Australia; samantha.nixon@uq.net.au (S.A.N.); z.dekan@imb.uq.edu.au (Z.D.); s.robinson@imb.uq.edu.au (S.D.R.); s.guo@imb.uq.edu.au (S.G.); i.vetter@imb.uq.edu.au (I.V.); p.alewood@imb.uq.edu.au (P.F.A.); 2 CSIRO Agriculture and Food, St Lucia, QLD 4072, Australia; Andrew.Kotze@csiro.au; 3 School of Pharmacy, The University of Queensland, Woolloongabba, QLD 4102, Australia; 4 School of Science & Engineering, University of the Sunshine Coast, Sippy Downs, QLD 4556, Australia

**Keywords:** ant, venom, venom peptide, pilosulin, heterodimer, antiparasitic, antimicrobial

## Abstract

Ant venoms have recently attracted increased attention due to their chemical complexity, novel molecular frameworks, and diverse biological activities. The heterodimeric peptide ∆-myrtoxin-Mp1a (Mp1a) from the venom of the Australian jack jumper ant, *Myrmecia pilosula*, exhibits antimicrobial, membrane-disrupting, and pain-inducing activities. In the present study, we examined the activity of Mp1a and a panel of synthetic analogues against the gastrointestinal parasitic nematode *Haemonchus contortus*, the fruit fly *Drosophila melanogaster*, and for their ability to stimulate pain-sensing neurons. Mp1a was found to be both insecticidal and anthelmintic, and it robustly activated mammalian sensory neurons at concentrations similar to those reported to elicit antimicrobial and cytotoxic activity. The native antiparallel Mp1a heterodimer was more potent than heterodimers with alternative disulfide connectivity, as well as monomeric analogues. We conclude that the membrane-disrupting effects of Mp1a confer broad-spectrum biological activities that facilitate both predation and defense for the ant. Our structure–activity data also provide a foundation for the rational engineering of analogues with selectivity for particular cell types.

## 1. Introduction

The Australian jack jumper ant, *Myrmecia pilosula*, is a species of bull ant within the *M. pilosula* species complex, endemic to the temperate Eastern regions of Australia [1]. These ants are well known for their jumping ability and highly painful stings that can cause severe allergic reactions [2,3]. *M. pilosula* venom is a cocktail of peptidic toxins (2–25 kDa) that is employed for both predation and defense [4]. By far the most abundant venom component is a small, antiparallel disulfide-linked heterodimeric peptide named Δ-myrtoxin-Mp1a (Mp1a) (previously referred to as ‘pilosulin 3’) [5,6,7]. 

Although most animal venoms have evolved to assist with predation, they are used for a variety of other roles including defense against predators (e.g., bees [8]), intraspecific competition (e.g., platypus [9]), conspecific communication (e.g., wasps [10]), chemical detoxification (e.g., formicine ants [11]), detection of envenomed prey (e.g., rattlesnakes [12]), and courtship and mating (e.g., scorpions [13]). Thus, Mp1a could play a number of roles in *M. pilosula* venom, but its ecological function is currently not clear. Intraplantar injection of Mp1a into mice induces nocifensive behavior and mechanical allodynia [5]. Mp1a also has broad-spectrum activity against a diverse range of bacteria, fungi and cell lines [5]. We hypothesized that Mp1a, as a membrane-disrupting peptide and the major venom component, might serve both defensive (i.e., pain-inducing) and predatory (i.e., insecticidal) roles.

There is also growing interest in ant venoms as a source of structurally and pharmacologically diverse peptides with potential applications in medicine, agriculture and biotechnology [14]. Mp1a and synthetic analogues have already been shown to have antibiotic activity, including against the opportunistic human pathogen *Acinetobacter baumannii* [5]. Several venom-derived antimicrobial peptides are active against a range of human parasites, including cupiennin 1a from the wandering spider *Cupiennius salei*, which is active against both trypanosomes and malaria parasites [15,16], the antimalarial peptides meucin-24 and 25 from the scorpion *Mesobuthus eupeus* [17], and the antitrypanosomal dinoponeratoxins from the giant ant *Dinoponera quadriceps* [18]. We therefore explored whether Mp1a might be active against *Haemonchus contortus*, a pathogenic blood-feeding parasitic nematode of ruminants, which serves as a model organism for anthelmintic drug discovery. While often found in ant venoms [14,19], small disulfide-bridged heterodimeric toxins are uncommon in nature. We therefore used a series of synthetic Mp1a analogues to explore the structure—activity relationships for Mp1a in relation to their insecticidal, cytotoxic, antiparasitic and algogenic properties to assess their selectivity and potential as human or veterinary therapeutics.

## 2. Experimental Section

### 2.1. Peptide Synthesis

The linear A- and B-chains of Mp1a were synthesized via Fmoc solid phase peptide synthesis (SPPS) and purified using reversed-phase high-performance liquid chromatography (RP-HPLC) [5]. Mp1a (**1a**) was formed by mixing the reduced forms of the two chains in an equimolar ratio at pH 8.0. The cysteine oxidation reaction yielded a single major product that co-eluted with the major venom toxin on RP-HPLC [5], indicating that correct disulfide pairings had been achieved and potential homodimers, cyclized monomers, parallel heterodimers or polymers were not formed [5]. The monomeric chains of Mp1a (**3a** and **4a**) were readily prepared by dimethyl sulfoxide (DMSO) oxidation. Analogue **5a** was produced using the purified reduced form of the A-chain dissolved in 6 M guanidium hydrochloride (GnHCl) and diluted with 0.2 M NH_4_HCO_3_ (pH 8.0) to a final concentration of 2 mM peptide and 1 M GnHCl, and then stirred in an open vessel at room temperature for 48 h. Analysis of the product using HPLC and MS revealed a predominant peak corresponding to the correct mass of the dimeric A chain (calc. avg. 1577.0 M + 4H, found 1576.9), which was isolated using preparative HPLC.

### 2.2. Drosophila Melanogaster Microinjection Assay

The insecticidal activity of the chemically synthesized **1a** and analogues thereof were determined by injection into adult female *D. melanogaster* aged 3–5 days (mass 0.7–0.9 mg) as described previously [20]. Analogues were dissolved in water and intrathoracically injected using pulled glass capillaries at a volume of 50 nL per fruit fly, and the results were compared against an equivalent injection volume of water alone. All injections were performed in the morning or early afternoon. At 24 h post-injection flies were monitored for lethality. Dose–response curves were constructed using 7–10 doses per analogue (each dose tested in eight flies) and three separate dose–response curves were constructed for each analogue. The lethality in the groups receiving the analogues were adjusted using the respective control group lethality using the Henderson-Tilton formula [20]. The median lethal dose (LD_50_) of each analogue was calculated based on the three dose–response curves using GraphPad Prism 8.0 [21]. One-way ANOVA followed by Dunnett’s multiple comparison test were used to compare LD_50_ values. 

### 2.3. Calcium Imaging of Mammalian Sensory Neurons

Calcium imaging of a heterogenous population of mouse dorsal root ganglion (DRG) sensory neurons was performed as previously described [22]. In brief, dorsal root ganglion (DRG) cells isolated from 4–8-week-old male C57BL/6 mice under ethics approval TRI/IMB/093/17 (University of Queensland Animal Ethics Committee, approval date 31/03/2017) were dissociated and then plated in Dulbecco’s modified Eagle’s medium (Gibco, Grand Island, NY, USA) containing 10% fetal bovine serum (FBS; Assaymatrix, Melbourne, Australia) and penicillin/streptomycin (Gibco) on a 96-well poly-D-lysine–coated culture plate (Corning, Lowell, MA, USA), and maintained overnight. Cells were loaded with Fluo-4 AM calcium indicator as per the manufacturer’s instructions (Thermo Fisher Scientific, Grand Island, NY, USA). After loading for 1 h, the dye solution was replaced with assay solution (Hanks’ balanced salt solution and 20 mM HEPES). Fluorescence corresponding to intracellular calcium ([Ca^2+^]_i_) of typically 100–150 DRG cells per experiment was monitored in parallel using a Nikon Ti-E deconvolution inverted microscope, equipped with a Lumencor Spectra LED light source. Images were acquired at a 20× objective at one frame/s (excitation 485 nm; emission 521 nm). For each experiment, baseline fluorescence was monitored for 30 s and then a wash of assay solution was applied. At 60 s, the assay solution was replaced with assay solution containing individual peptides (10 µM) and the cells were observed for a further 90 s.

### 2.4. FLIPR Assay 

F11 cells were cultured as previously described [23]. Cells were maintained on Ham’s F12 media supplemented with 10% fetal bovine serum (FBS), 100 µM hypoxanthine, 0.4 µM aminopterin, and 16 µM thymidine (Hybri-MaxTM, Sigma Aldrich, North Ryde, Australia). Then, 384-well imaging plates (Corning) were seeded 48 h prior to imaging resulting in 90–95% confluence at imaging. Cells were incubated for 30 min with the Calcium 4 assay component A according to the manufacturer’s instructions (Molecular Devices, Sunnyvale, CA, USA) in physiological salt solution (PSS; composition in mM: 140 NaCl, 11.5 D-glucose, 5.9 KCl, 1.4 MgCl_2_, 1.2 NaH_2_PO_4_, 5 NaHCO_3_, 1.8 CaCl_2_, 10 HEPES) at 37°C. Fluorescence was measured using a fluorescent imaging plate reader (FLIPR^TETRA^) equipped with a CCD camera (Excitation: 470–490 nm, Emission: 515–575 nM) (Molecular Devices, Sunnyvale, CA, USA). Signals were read every second for 10 s before, and 300 s after the addition of peptides in PSS supplemented with 0.1% bovine serum albumin. All data are the mean ± SEM of assays performed in triplicate. Maximum–minimum fluorescence in the 300 s period after peptide addition was recorded as the response. Concentration–response data were fitted with a four-parameter Hill equation (variable slope) using GraphPad Prism 8 to obtain effective concentration (EC_50_) values.

### 2.5. Haemonchus Contortus Isolation and Larval Development Assay

Sheep were infected with *H. contortus* Kirby isolate (field isolate from the University of New England Kirby Research Farm in 1986; susceptible to all commercial anthelmintics [24]) and housed at the Commonwealth Scientific and Industrial Research Organisation (CSIRO) FD McMaster Laboratory, Armidale, NSW. All animal procedures were approved by the FD McMaster Animal Ethics Committee, CSIRO (Approval Number AEC 17/12, approval date 15.6.2017). Eggs were prepared from overnight fecal collection as described [25]. In brief, feces were filtered through mesh filters, settled, and supernatant removed by vacuum. Eggs were recovered by density centrifugation using 10 and 25% (*w*/*v*) sucrose solutions, centrifuged at 650× *g* for 7 min. Eggs were recovered from the interface of the two sucrose layers, rinsed with distilled water, sterilized with bleach, rinsed again, and diluted to 4500 eggs/mL. Tylosin tartrate (800 μg/mL) and amphotericin B (25.0 μg/mL) were added, and eggs were used immediately for larval development assays.

Assays were conducted using 96-well microtitre plates, with each well containing 50 μL of 2% agar, 20 μL of egg solution and 20 μL of peptide solution in water. The commercial anthelmintic levamisole (Sigma Aldrich) was used as a positive control (final 1% *v*/*v*). Negative controls contained equivalent volumes of water or DMSO (final 1% *v*/*v*). Plates were incubated at 26 °C for six days. After 24 h, each well was fed with 10 μL of a nutrient solution containing *Escherichia coli* XL1-Blue1 (grown overnight at 37 °C) and growth medium. The growth medium consisted of yeast extract (1% *w*/*v*), Earle’s salt solution (10% *v*/*v*), saline solution (0.9% NaCl, *w*/*v*), and sodium bicarbonate (1 mM) in Luria-Bertani medium (LB). Larvae were killed and stained with Lugol’s iodine solution after six days. Larvae that had developed to the infective L3 stage were counted, and the numbers in treated assay wells were expressed as a percentage of the number of infective L3 stage larvae in multiple control wells. Concentrations that caused 50% inhibition (IC50 values) were calculated from three experiments of duplicate assays. A biphasic response was observed for one peptide, and hence separate IC_50_ values were calculated for the two components of the response curve.

### 2.6. Cytotoxicity Assay 

HEK293 (ATCC^®^ CRL-1573, Gaithersburg, MD, USA) cells were seeded at 3000 cells/well in clear bottom 384-well plates (Corning) in a volume of 20 μL in DMEM medium (GIBCO-Invitrogen #11995-073, Grand Island, NY, USA) with 10% FBS. Cells were incubated at 37 °C in 5% CO_2_ for 24 h to allow cell attachment. Compounds were dissolved in water at 1.28 mg/mL with subsequent threefold dilutions in cell culture medium, giving a final concentration range of 0.02–50 μg/mL. Tamoxifen (Sigma-Aldrich) was used as a negative cell survival control as a single point 100 μM and as a dose response with concentrations ranging from 0.18 to 400 μM. The cells were incubated with the compounds for 24 h at 37 °C, 5% CO_2_. After incubation, 10 μM resazurin (Sigma-Aldrich, dissolved in PBS) was added to each well and the plates were incubated for a further 3 h at 37 °C, 5% CO_2_. The fluorescence intensity was measured using a Polarstar Omega (BMG Technologies, Mornington, Australia) spectrophotometer (BMG Labtech, Mornington, Australia) with excitation/emission of 560/590 nm. The concentration required to induce 50% cell death (CC_50_, determined as 50% reduction in absorbance relative to untreated control) was calculated using Graph Pad Prism 8.0. Cytotoxicity assays were performed as two independent experiments of duplicate assays to obtain data of *n* = 4.

### 2.7. Hemolysis Assay

Whole human blood (10 mL/tube) was washed in triplicate with three volumes of 0.9% NaCl, with centrifugation at 500× *g* (with reduced deceleration) for 10 min between washes. Cells were counted with a hemocytometer and then diluted to 0.5 × 10^8^/mL in 0.9% NaCl. Cell suspension (180 μL/well) was added to assay plates and then plates were sealed and incubated at 37 °C for 1 h. Plates were then centrifuged at 1000× *g* for 10 min to pellet cells and debris and 25 μL of supernatant was recovered into a 384-well flat bottom polystyrene plate. Absorbance was read at 405 nm using a Tecan M1000 Pro monochromator plate reader. The percentage of hemolysis was calculated for each well relative to the negative control (1% DMSO in PBS) and positive control (1% Triton X-100 in PBS). Significant differences in hemolysis values were determined by fractional deviation from the mean, calculated using the average and standard deviation of the sample wells (no controls) on the same plate. The concentration required to lyse 50% of the red blood cells (HC_50_) was determined from two independent experiments of duplicate assays to obtain data of *n* = 4.

### 2.8. Minimum Inhibitory Concentration (MIC) Dilution Assay

Compounds were serially diluted in cation-adjusted Mueller Hinton Broth (CaMHB) twofold across the wells of non-binding surface 96-well plates (Corning), plated in duplicate. Bacteria (strains listed in Table 1) were cultured in CaMHB at 37 °C overnight, then diluted 40-fold and incubated at 37 °C for a further 2–3 h. The resultant mid-log phase cultures were diluted in CaMHB and added to each well of the compound-containing 96-well plates to give a final cell density of 5 × 105 CFU/mL, and a final compound concentration range of 0.06–128 μg/mL. The plates were covered and incubated at 37 °C for 20 h. Inhibition of bacterial growth was determined visually, where the minimum inhibitory concentration (MIC) was recorded as the lowest compound concentration that yielded no visible growth. 

### 2.9. Statistical Analysis 

Significant differences in the insecticidal, anthelmintic and algogenic activities of Mp1a and analogues were determined using one-way ANOVA with multiple comparison followed by Tukey’s post-hoc test in GraphPad Prism 8.0. 

## 3. Results

### 3.1. Synthesis of Mp1a and Analogues

**Figure 1:**
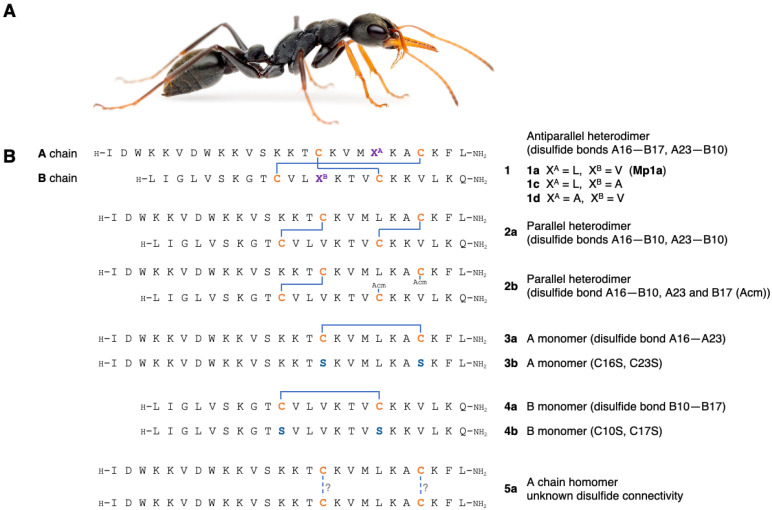
(**A**) Adult female *M. pilsoula* (photograph credit: Dr Alexander Wild). (**B**) Sequence and disulfide-bond connectivity of native ∆-myrtoxin-Mp1a (Mp1a) (**1a**) and synthetic analogues (**1a**–**4b** previously described [5]). The native peptide forms an antiparallel heterodimer comprised of one A chain and one B chain connected by two disulfide bonds (A16–B17, A23–B10). Cysteines are shown in orange, with blue lines indicating disulfide bonds. Dashed blue lines indicate unknown disulfide connectivity. Amino acid substitutions are coloured (serine in blue, miscellaneous in purple). Acetamidomethylated cysteine (Acm). Methylated cysteine (Me).

We synthesized Mp1a (**1a**) and analogues [5], as well as a novel A-chain homodimer with unknown disulfide connectivity (**5a**) (sequences and connectivity shown in Figure 1). Mp1a A and B-chains were produced in their reduced forms using Fmoc SPPS. Mp1a (**1a**) was formed exclusively as the antiparallel heterodimer by air oxidation of the two reduced chains without the use of orthogonal cysteine protecting groups. As no parallel heterodimer was obtained using this method, it was necessary to employ a directed strategy to prepare the non-native parallel heterodimers **2a** and **2b** as previously described [5]. 

### 3.2. Mp1a Activates Sensory Neurons

**Figure 2:**
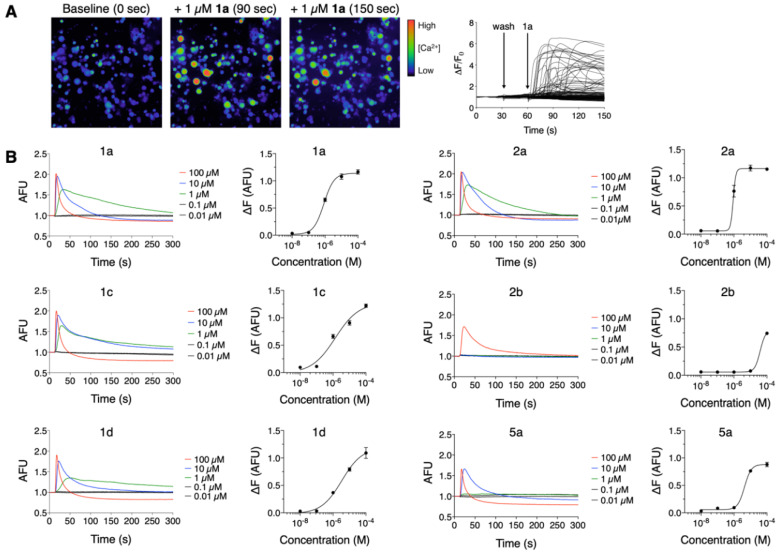
(**A**) Application of 1 µM Mp1a (**1a)** to dorsal root ganglion (DRG) cells induced a rapid increase in [Ca^2+^]_i_ followed by release of fluorescent dye into the media. The left panels (20x) show micrographs taken at baseline (0 s) and after addition of 1 µM **1a** (synthetic Mp1a. at 90 and 150 s). The graph on the right (20x) shows fluorescence responses for individual cells within the field of view. (**B**) Fluorescence responses of F11 cells after addition of **1a**–**1d**, **2a**, **2b** and **5a** over a range of concentrations (0.01–100 µM) recorded using a FLIPR. Changes in fluorescence (∆F) from the maximal response less baseline fluorescence were recorded (*n* = 3) and used to generate concentration–response curves.

Mp1a induces spontaneous pain and mechanical allodynia when injected into the hind paw of mice [5]. We used calcium imaging to investigate the effects of Mp1a (**1a**) on mouse DRG sensory cells, and to assess how structural modifications affect cellular activation. Stimulation of nociceptive neurons can be initiated via activation of various ion channels or receptors, but it is invariably associated with an increase [Ca^2+^]_i_ in these neurons resulting from downstream activation of voltage-gated calcium (Ca_V_) channels. Thus, increases in [Ca^2+^]_i_ in sensory neurons can be used as a proxy for activation [22,26,27]. The addition of 1 µM **1a** resulted in a rapid increase in [Ca^2+^]_i_ in all cells (both neuronal and non-neuronal). This was followed by a gradual decrease in fluorescence as dye leaked into the extracellular medium, indicating cytolysis [22] (Figure 2A). The heterodimeric analogues (**1**–**2**) and the A-homodimer **5a** also activated all DRG cells and caused dye leakage; however, at 1 µM, analogues **3**–**4** had no effect.

The activity of the peptides was quantified via FLIPR analysis of the effect on F11 cells (a neuroblastoma × DRG neuron hybrid cell line) (Figure 2B). The effective concentration (EC_50_) for stimulation of these cells ranged from 0.8 to 5 µM, with the exception of **2b** which was 45-fold less active than native Mp1a (EC_50_ 38.5 µM). In F11 cells, all peptides induced a rapid increase in fluorescence followed by a decrease to or below baseline, reflecting cytolysis. This effect was observed at concentrations ≥ 1 µM for **1a**, **1c**, **1d**, and **2a**, ≥ 10 µM for **5a,** and 100 µM for **2b** (Table 2). 

### 3.3. Synthetic Mp1a Shows Insecticidal Activity against D. melanogaster 

**Figure 3:**
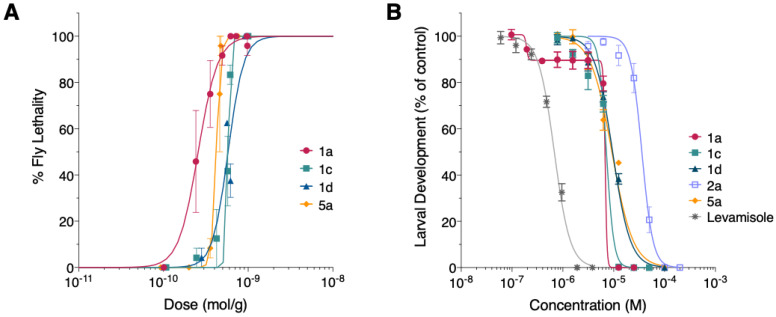
(**A**) Dose–response curves for insecticidal effects of Mp1a analogues **1a**, **c**, **d** and **5a** following microinjection into adult female fruit flies (*D. melanogaster*). (**B**) Concentration–response curves for anti-parasitic effects of Mp1a analogues against *H. contortus*. For both assays, data points represent the mean ± standard error of the mean (SEM) based on *n* = three experiments.

We used a *D. melanogaster* injection model to investigate the insecticidal activity of Mp1a, as *M. pilosula* favours predation of smaller fly species [28]. The injection of synthetic Mp1a (**1a**) resulted in a rapid paralysis of the flies that was ultimately lethal over a 24 h period with an LD_50_ of 260 pmol/g (Figure 3A). Of the analogues, only the antiparallel heterodimers **1c** and **1d** and the A chain homodimer **5a** were insecticidal, but they were less active (LD_50_ values of 415–578 pmol/g) than the native heterodimer **1a** (Table 2). All other analogues were inactive in fruit flies, with LD_50_ values > 600 pmol/g, indicating that the antiparallel heterodimeric scaffold is important for the insecticidal activity of Mp1a.

### 3.4. Mp1a Is Active against the Gastrointestinal Nematode H. contortus In Vitro 

We investigated whether Mp1a and analogues could inhibit the larval development of *H. contortus*. Analogue **1a** and the native-like antiparallel heterodimers **1c** and **1d**, and the A-chain homodimer **5a**, inhibited larval development with IC_50_ values of 6.8–9.5 µM, approximately 10-fold higher than the commercial anthelmintic levamisole (Figure 3B). One-way ANOVA indicated that the anthelmintic activities of **1a**–**1d** and **5a** were not significantly different from each other. In contrast the parallel homodimer (**2a**) was fivefold less active and all other analogues were significantly less active against the parasite (between six and 10-fold, exemplified by **2a** in Figure 3B, rest not shown, *p* < 0.001). Analogue **1a** showed a biphasic response, with larval development reduced to 90% of that observed in control assays at low micromolar concentrations (< 2 µM), followed by the complete inhibition of development at higher peptide concentrations. The IC_50_ value for the response observed at low concentrations was 0.19 µM, compared to the IC_50_ of 6.8 µM observed at higher concentrations. This biphasic response was not observed with other analogues. 

### 3.5. Cytotoxicity and Antimicrobial Activity 

Analogues 1–4 were previously assayed for cytotoxicity and antimicrobial activity [5]. We screened the novel A chain homomer **5a** against the human embryonic kidney-derived cell line HEK293 and human red blood cells. Analogue **5a** was cytotoxic to HEK293 cells (CC_50_ 2.2 µM) but was less active against red blood cells (HC_50_ > 10 µM, Table 1). Analogue **5a** also had low micromolar activity against Gram-negative and Gram-positive bacterial pathogens and was most active against the important human pathogen *A. baumannii* (MIC 0.03—0.1 µM, Table 1). 

## 4. Discussion

Recent advances in omics technologies have enabled detailed study of venoms produced only in small quantities, such as those of ants. This has revealed an unsuspected diversity of ant venom peptides [7,8,19] and has increased interest in the potential of ant venom peptides for development as biomedicines and pharmacological tools [29]. Such developments would be supported by further knowledge on the structure—activity relationships of ant toxins. We investigated the bioactivity of the antiparallel heterodimeric venom peptide Mp1a from the Australian jack jumper ant, *M. pilosula*, to explore its ecological role and to assess its potential use as a veterinary or human therapeutic. We report that synthetic Mp1a (**1a**) has broad-spectrum bioactivity, which includes moderate insecticidal activity, potent anthelmintic activity against the veterinary nematode *H. contortus*, and the robust activation of mammalian sensory neurons (summarised in Table 2).

### 4.1. Mp1a Shows Insecticidal and Algogenic Activities, Dependent on Dimerization

Injection of Mp1a into fruit flies resulted in rapid, irreversible paralysis leading to death. *M. pilosula* is reported to preferentially prey on small flies [28], so this finding is consistent with Mp1a serving a predatory function. The insecticidal potency of Mp1a is similar to that of the spider toxins ω/κ-hexatoxin-Hv1c (LD_50_ 210 pmol/g) and U_1_-agatoxin-Ta1a (240 pmol/g), but lower than the highly potent insecticidal spider toxins ω-hexatoxin-Hv1a (9 pmol/g) and β-diguetoxin-Dc1a (59 pmol/g), in the same assay [20]. Native Mp1a was the most active of the ant peptides tested, with the antiparallel analogues **1c** and **1d** being significantly less active (LD_50_ ~550 pmol/g, *p* < 0.0001) and the A-chain homomer **5a** wa**s** 1.5-fold less active (LD_50_ 415 pmol/g, *p* < 0.002). All remaining analogues had LD_50_ values well over 600 pmol/g, indicating that the heterodimer and antiparallel orientation are important for insecticidal activity. We hypothesize that due to Mp1a’s membrane-disrupting effects, this insecticidal activity likely extends to other insect species, though this remains to be tested.

The addition of low micromolar concentrations of Mp1a to cells isolated from mouse dorsal root ganglia resulted in the rapid activation of both neuronal and non-neuronal cells. This was followed by a decrease in [Ca^2+^]_i_ reflective of cytolysis (Figure 2a). These data are consistent with Mp1a’s previously reported membrane-disrupting activity and algogenic effects in mice after intraplantar injection [5]. Similar effects were observed from each dimeric peptide analogue but, in striking contrast, the monomers were inactive up to 10 µM. Analogue **1a** activated cells at submicromolar concentrations (EC_50_ 0.85 µM) followed by rapid lysis. Analogues **1c**, **1d** and **2a** similarly activated cells at low micromolar concentrations, with **5a** being fivefold less active than **1a**. These data indicate that the dimerization of Mp1a is also critical for its algogenic activity and thus its presumed defensive role in the venom.

### 4.2. Mp1a Shows Antiparasitic Activity against the Veterinary Nematode H. contortus

Antimicrobial peptides with membrane-disrupting and cytolytic activities, including those from animal venoms, have previously been shown to be antiparasitic [15]. However, very few studies have reported venom peptides with activity against nematode parasites [29,30], and none from ants. *H. contortus* is a highly virulent gastrointestinal ruminant that shows widespread drug-resistance [31], and recent reports of its multidrug resistance [32] have highlighted the need for new treatments. This prompted us to screen Mp1a and its analogues for their antiparasitic activity against *H. contortus*. Synthetic Mp1a (**1a**) was found to have low micromolar anthelmintic activity against the larval stages of the nematode (6.8 µM). Analogue **1a** is about threefold more potent than the previously reported anthelmintic activity of the spider-venom peptide Hi1a in the same assay (IC_50_ 22.9 µM) [30], but 10-fold less active than the commercial anthelmintic levamisole (IC_50_ 0.68 µM, Figure 3B) in a drug-susceptible isolate. We hypothesize that Mp1a’s broad-spectrum cytolytic activity will extend its anthelmintic activity to drug-resistant isolates, but this remains to be confirmed. 

Interestingly, **1a** showed a biphasic response in the larval development assay (Figure 3B), which was not observed in any other analogues. One possible explanation for this phenomenon is that a small fraction of the nematodes (~10%) had greater sensitivity to the toxin. A biphasic concentration–response curve was previously observed in a monepantel-resistant isolate of *H. contortus*, with the larval development assay revealing the presence of two distinct sub-populations showing low and high levels of resistance to the anthelmintic [33]. Alternatively, there may be a secondary molecular target for Mp1a. The cause of this biphasic response remains an area for further investigation. 

The other antiparallel heterodimeric analogues **1c** and **1d,** as well as the A-chain homodimer **5a,** also showed low micromolar activity against nematodes, indicating that dimerization is important for anthelmintic activity. However, the anthelmintic activity of the dimers was only observed at concentrations 10-fold higher than required for neuronal cell activation, cytotoxicity and hemolysis. Analogues with reduced cytotoxicity, such as the parallel heterodimers **2a** and **2b**, were also less active against *H. contortus*, suggesting that the anthelmintic activity is likely due to non-specific cytolytic activity. This close link between anthelmintic activity, cytotoxicity and sensory neuron activation suggests that these analogues would also have adverse effects in vivo, similar to the known nocifensive activity of Mp1a and would, for this reason, make poor antiparasitic drug candidates. This contrasts with the broadly cytolytic peptide cupiennin-1a from the wandering spider, *C. salei*, which has > 400-fold selectivity for trypanosomes over human red blood cells [15]. Thus, further investigation into venom-derived membrane-disrupting peptides may still enable the identification or engineering of more suitable antiparasitic drug leads.

### 4.3. Mp1a Analogues Show Some Selectivity Across Bioassasys

In general, we found that the bioactivities across all assays were correlated, with the dimerization of Mp1a being critical for its insecticidal, anthelmintic and algogenic activities. Some analogues had better taxonomic selectivity; for example, the A-chain homodimer **5a** was fourfold less active than **1a** against HEK293 and red blood cells (Table 1), though this was still closely correlated with both insecticidal and anthelmintic activity. The A and B-chain monomers were essentially inactive across all assays, consistent with previous studies of Mp1a’s antimicrobial activity [5]. Interestingly, the native-like antiparallel orientation of the A and B-chains was important for both anthelmintic and insecticidal activity; the parallel-oriented heterodimers **2a** and **2b** were four- to eightfold less active against *H. contortus* and completely inactive against *D. melanogaster* (Table 1). In contrast, parallel heterodimer **2a** was equipotent with synthetic Mp1a **1a** against F11 cells in a FLIPR model of sensory neuron activation, suggesting that chain orientation may be less important in neuronal membrane interactions. The exception was single disulfide-bridged heterodimer **2b** which was 45-fold less active than the native heterodimer (EC_50_ 38.5 µM, *p* < 0.0001) in the sensory neuron assay (Table 1). This peptide was similarly less active against *H. contortus* and inactive against *D. melanogaster* (LD_50_ > 600 pmol/g), which may be due to its increased flexibility because of the single disulfide bridge. Promisingly, however, this peptide has a potent activity against the Gram-negative pathogen *A. baumannii* (minimum inhibitory concentration 0.025–0.1 µM), approximately 1500-fold lower than its EC_50_ against F11 cells. These data suggest that there may be opportunities to increase the selectivity of Mp1a analogues as antimicrobials. One unexplored analogue is the B-chain homodimer, as the A-homodimer **5a** had a similar insecticidal and anthelmintic activity as the native-heterodimer **1a** (1.2- and 1.5-fold changes respectively). Future structure–activity relationship studies could explore further modifications such as N-terminal acylation, which was found to increase the affinity of the wasp venom peptide mastoparan-X for negatively charged bacterial membranes, thereby improving selectivity over host cells [34].

Based on the present and previously reported data, Mp1a shows a remarkably broad-spectrum bioactivity, with insecticidal, algogenic, cytotoxic, hemolytic, and antimicrobial activities that rely upon the dimerization of the peptide. Thus, Mp1a seems to serve both predatory and defensive roles in *M. pilosula* venom. The multifunctional nature of Mp1a may explain why it is the predominant toxin in the relatively simple *M. pilosula* venom, in contrast with spider [35], scorpion [36] and cone snail [37] venoms, which contain a rich diversity of ion channel-modulating toxins.

## Figures and Tables

**Table 1:** Minimum inhibitory concentrations of **5a** (A-chain homomer) and **1a** (native antiparallel heterodimer) against Gram-negative and Gram-positive bacteria from the genera *Escherichia*, *Klebsiella*, *Acinetobacter*, *Pseudomonas*, and *Staphylococcus*.

Strain	MIC 1a (µM) ^a^ (Native Antiparallel Heterodimer)	MIC 5a (µM) (A-Chain Homomer)
*E. coli* ATCC 25922	0.1–0.2	0.42–0.84
*K. pneumoniae* ATCC 700603	0.4–0.8	0.84–1.67
*K. pneumoniae* ATCC BAA-2146	0.1–0.2	0.84
*A. baumannii* ATCC 19606	0.025	0.03–0.1
*P. aeruginosa* ATCC 27853	0.4–0.8	0.84
*P. aeruginosa* FADDI-PA70	0.8	0.84–3.34
*S. aureus* ATCC 43300	0.8	1.67–3.34
*S. aureus* NRS 1	3.2	6.7
*S. aureus* NRS 17	0.4–0.8	1.67
*S. aureus* NARSA-VRS1	3.2–6.4	> 6.7
*S. aureus* NARSA-VRS10	0.8	1.67
*S. pneumoniae* ATCC 700677	0.4–0.8	1.67

^a^ Minimum inhibitory concentration (MIC) (µM) values for **1a** taken from Dekan et al. [5].

**Table 2:** Summary of bioassay data for synthetic Mp1a and analogues, including effective concentration (EC_50_) for activation of F11 cells, concentrations that caused 50% inhibition (IC50) for inhibition of larval development of *H. contortus*; median lethal dose (LD_50_) for lethal insecticidal effects on *D. melanogaster*; concentrations required to induce 50% cell death (CC_50_) for effects on viability of HEK293 cells, and concentrations required to lyse 50% of the red blood cells (HC_50_) for lysis of human red blood cells. All errors are SEM.

Peptide	Description	Neuronal Cell Activation (EC_50_, µM)	Anthelmintic Activity (IC_50_, µM)	Insecticidal Activity (LD_50_, pmol/g)	Cytotoxicity (CC_50_ µM) ^b^	Hemolysis (HC_50_ µM) ^b^
**1a**	Antiparallel heterodimer (native)	0.85 ± 0.03	6.8 ± 0.5 ^c^	260.1 ± 16.6	0.6	2.2
**1c**	Antiparallel heterodimer	1.39 ± 0.34	7.2 ± 0.1	552.7 ± 18.2 ^a^	0.7	> 12
**1d**	Antiparallel heterodimer	3.38 ± 2.8	9.5 ± 0.3	578 ± 9.7 ^a^	0.7	> 12
**2a**	Parallel heterodimer	0.9 ± 0.08	35.8 ± 1.9 ^a^	> 600	> 10	> 10
**2b**	Parallel heterodimer	38.5 ± 2.3 ^a^	65.6 ± 14.7 ^a^	> 600	> 10	> 10
**3a**	A monomer (A16–A23)	inactive	46.8 ± 0.6 ^a^	> 600	> 20	> 20
**3b**	A monomer [C16S, C23S]	inactive	64.9 ± 6.3 ^a^	> 600	> 20	> 20
**4a**	B monomer (B10–B17)	inactive	65.5 ± 5.5 ^a^	> 600	> 15	> 15
**4b**	B monomer [C10S, C17S]	inactive	61.3 ± 4.4 ^a^	> 600	> 15	> 15
**5a**	A chain homomer	4.3 ± 1.3	9.2 ± 0.7	415.4 ± 29.6 ^a^	2.4	> 10

^a^ Significantly different relative to **1a** based on one-way ANOVA. ^b^ Data from Dekan et al. [5] with the exception of analogue **5a**. ^c^ IC_50_ value shown here represents the response observed in the majority of the nematode population (approximately 90%). The IC_50_ value for the response observed at lower concentrations (< 2 µM) was calculated at 0.2 ± 0.1 µM.
